# Allergy-specific Phenome-Wide Association Study for Immunogenes in Turkish Children

**DOI:** 10.1038/srep33152

**Published:** 2016-09-14

**Authors:** Sefayet Karaca, Ersoy Civelek, Mehmet Karaca, Umit M. Sahiner, Riza K. Ozgul, Can N. Kocabas, Renato Polimanti, Bülent E. Sekerel

**Affiliations:** 1 Aksaray University, Faculty of Health Science, Aksaray, Turkey; 2 Ankara Child Health and Diseases Hematology Oncology Research Hospital, Pediatric Allergy and Immunology Clinic, Ankara, Turkey; 3 Aksaray University, Faculty of Science and Arts, Department of Biology, Aksaray, Turkey; 4 Hacettepe University, Faculty of Medicine, Pediatric Allergy and Asthma Unit, Ankara, Turkey; 5 Hacettepe University, Faculty of Medicine, Pediatrics Department, Unit of Metabolism and Institute of Child Health, Ankara, Turkey; 6 Department of Psychiatry, Yale University School of Medicine and VA CT Healthcare Center, West Haven, CT, United States

## Abstract

To dissect the role of immunogenetics in allergy and asthma, we performed a phenome-wide association study in 974 Turkish children selected from a cross-sectional study conducted using ISAAC (International Study of Asthma and Allergies in Children) Phase II tools. We investigated 9 loci involved in different immune functions (*ADAM33*, *ADRB2*, *CD14, IL13*, *IL4*, *IL4R*, *MS4A2*, *SERPINE1*, and *TNF*) with respect to 116 traits assessed through blood tests, hypertonic saline challenge tests, questionnaires, and skin prick tests. Multiple associations were observed for *ADAM33*: rs2280090 was associated with reduced MEF240% (i.e., the ratio of Mean Expiratory Flow after 240s of hypertonic saline inhalation with respect to the age- and ancestry-matched reference value) and with an increased risk of allergic bronchitis (p = 1.77*10^−4^ and p = 7.94*10^−4^, respectively); rs3918396 was associated with wheezing and eczema comorbidity (p = 3.41*10^−4^). *IL4* rs2243250 was associated with increased FEV240 (Forced Expiratory Flow Volume after 240s of hypertonic saline inhalation; p = 4.81*10^−4^) and *CD14* rs2569190 was associated with asthma diagnosis (p = 1.36*10^−3^). *ADAM33* and *IL4* appeared to play a role in the processes linked to allergic airway inflammation and lung function. Due to its association with wheezing and eczema comorbidity, *ADAM33* may also be involved in the atopic march.

Risk alleles located in genes involved in immune systems and functions have been established by genome-wide association studies (GWAS)^1^,^2^, confirming the pivotal role of the immunogenetics in the predisposition to asthma and other allergic respiratory diseases. In particular, the genetic basis of the immune response has been demonstrated to be involved in the childhood onset of allergic respiratory diseases^3^. Although GWAS is a powerful tool to investigate the genetic architecture of complex traits, the number of variants identified is proportional to the sample size of the cohorts investigated. To date, a relatively restricted number of loci have been confirmed by GWAS of allergic respiratory diseases, suggesting that other risk loci are still missing. This is likely due to the fact that the predisposition to complex traits is highly polygenic^4^ and huge sample size are necessary to identify a large portion of the risk loci associated. Beyond GWAS, other methods have been proposed to study the genetics of complex traits. Phenome-wide association studies (PheWAS) have recently been proven to identify novel phenotypic associations of previously identified risk loci^5^,^6^. Accordingly, a PheWAS focused on a wide range of phenotypic traits involved in a specific disease category can confirm risk loci previously identified by molecular experiments that have not yet been confirmed by GWAS.

In the present study, we conducted a PheWAS for immunogenes (i.e., genes involved in the immune system and its functions) in 974 Turkish children from a cross-sectional study conducted using the ISAAC (International Study of Asthma and Allergies in Children) Phase II tools. We analyzed 116 traits related to allergy and respiratory diseases that were assessed through blood tests, bronchial challenge tests, questionnaire evaluation, and skin prick tests. Nine immunogenes (*ADAM33*, *ADRB2*, *IL13*, *IL4*, *IL4R*, *MS4A2*, *CD14*, *SERPINE1*, and *TNF*) were selected for this analysis on the basis of their consistent connection to immune functions and allergy- and asthma-related phenotypes^7^.

*ADAM33* is involved in a variety of biological processes related to cell-cell and cell-matrix interactions, including fertilization, muscle development, neurogenesis, asthma, and allergy^8^. *ADRB2* encodes beta-2-adrenergic receptor that is associated with nocturnal asthma, obesity and type 2 diabetes^9^. *CD14* protein product is a membrane glycoprotein of T lymphocytes that interacts with major histocompatibility complex class II antigens^10^. *IL13* codifies an immunoregulatory cytokine that is involved in B-cell maturation and differentiation and pro-inflammatory cytokine and chemokine inhibition^11^. *IL4* encodes a cytokine produced by activated T cells that is involved in a wide range of activities^11^. *IL4R* protein product is the interleukin 4 receptor bound by both *IL13* and *IL4*^12^. *MS4A2* gene codifies the beta subunit of the high-affinity IgE receptor, involved in allergy and parasites immunity^13^. *SERPINE1* codifies the plasminogen activator inhibitor-1, which is a member of the serine proteinase inhibitor superfamily^14^. *TNF* gene product is a multifunctional proinflammatory cytokine that is involved in autoimmune diseases, insulin resistance, and cancer^15^.

To our knowledge, this is the largest genetic association study considering allergy-asthma related phenotypes in a Turkish cohort. This study contributed to understanding the genetics of asthma- and allergy-related phenotypes in non-European individuals in order to reduce health disparities among human populations. Indeed, the Turkish population is an admixture of European, Middle Eastern, and Central Asian ancestries and previous studies have confirmed that genetics contributes to the epidemiological differences observed between Turkish and European subjects^16^,^17^,^18^,^19^. In particular, comparing Turkish and European populations, we identified functional haplotype diversity of the immunogenes investigated between Turkish and northern/western European populations^20^.

## Materials and Methods

### Subjects

All procedures used in this study conform to the tenets of the Declaration of Helsinki and received approval from the Ethical Review Board of Ankara Child Health and Diseases Research Hospital. The subjects analyzed in the present study (n = 974) were selected from a cross-sectional study conducted using the ISAAC Phase II tools on schoolchildren from different city centers (Van, Manisa, Ankara, Antalya, and Trabzon) located in five regions of Turkey (East, Aegean, Central Anatolian, Mediterranean and Black Sea regions, respectively) between September 15, 2005 and May 30, 2006^21^. Written informed consent was obtained from all the participant.

All of the questions included in the ISAAC Phase II modules were included in the data collection^22^. A pre-existing translation of the questionnaire was used, with visual improvements and minor changes to the question ordering made^23^. According to the ISAAC protocol, laboratory tests including blood tests, bronchial challenge tests, and skin prick tests were performed. The blood tests were conducted in the central laboratories of the five participating university hospitals in the respective cities using standard protocols. The bronchial challenge test was conducted with hypertonic saline using a De Vilbiss ultrasonic nebulizer (De Vilbiss, Langen, Germany) and the ZAN100 Spirometry System (nSpire Health, Longmont, Colorado, USA) in accordance with the recommended method^22^. The skin prick tests were performed using a multi-prick test device (Quantitest, Panatrex Inc, Placentia, California, USA) on the volar surface of both forearms, with results recorded after 15 minutes. Considering all phenotypic information collected, we extracted 116 allergy-relevant traits to be tested in the PheWAS (Supplemental Table 1). The main characteristics of the study population are reported in the Table 1. Further details regarding the sampling and phenotype assessments and comorbidity and phenotypic correlation in this cohort are available in our previous publications^21^,^24^,^25^,^26^.

### Genotyping procedures

DNA samples of participants were gathered from the project (A Multicenter Study, to Estimate the Prevelance of Childhood Allergic Diseases in Turkey: ISAAC Phase II. 03K120570-05-7) supported by the State Planning Organization of Turkey, during 2005-2006. Sequence-specific amplification primers (Supplemental Table 2) were designed commercially (LGC Genomics). We analyzed common variants with functional effects for the nine immunogenetic loci investigated: *ADAM33* (rs2787094, rs543749, rs2280090, rs2280091, rs3918396, rs6127096, and rs511898), *ADRB2* (rs1042713 and rs1042714), *CD14* (rs2569190), *IL13* (rs1800925, rs1295686, and rs20541), *IL4* (rs2243250 and rs2070874), *IL4R* (rs1805015, rs1801275), *MS4A2* (rs1441586, rs569108), *SERPINE1* (rs1799768), and *TNF* (rs1800629). Details regarding functional evidences related to the variants investigated were reported in our previous study^20^. Genotyping was performed using the Competitive Allele Specific PCR (KASP) technique^27^. 5–10ng of DNA were used per well, and PCR reactions were carried out in the presence of positive (samples with known genotypes) and no-template-controls (NTCs), at a 10 μl final volume (GeneAmp PCR System 9700, AppliedBiosystems). The KASP master mix, assay mix, and cycling conditions were based on manufacturers’ protocols (available at http://www.kbioscience.co.uk). Amplification products were read using a FRET-capable plate reader (FLUOstar Omega, BMG-Labtech), and KlusterCaller™ software was used to view genotyping data. For all variants analyzed, missing genotypes were <5% and the genotype frequencies were in Hardy-Weinberg equilibrium (exact test^28^ p > 0.05). Details regarding genetic quality control parameters are reported in Supplemental Table 3.

### Statistical Analysis

Plink 1.07^29^ was used to implement logistic and linear regression analyses for the association between genetic variants (additive model) and phenotypic traits (binary and quantitative, respectively). Quantitative traits were normalized using appropriate Box-Cox power transformations before being entered into the analysis. We adjusted the association analysis considering three covariates: age, sex, and sampling center. Since PheWAS are not discovery studies (they are follow up investigations useful to dissect the role of previously identified loci in human phenome), they can be correct with less stringent multiple testing criteria than the Bonferroni correction for the number of independent tests. In our study, we adjusted our results using a locus-wise Bonferroni correction (which corresponds to correction for the number of phenotypes) that was recently proposed by Simonti and colleagues^30^. We applied the matSpD method (available at http://neurogenetics.qimrberghofer.edu.au/matSpD/) to determine the effective number of independent phenotypes^31^. We opted to use this multiple comparison approach because of the high correlation among the allergy phenotypes investigated. We calculated that the phenome-wide significance (PWS) threshold to keep the type I error rate at 5% is p = 7.53*10^−4^. We also considered p = 1.47*10^−3^ to be the suggestive significance threshold (type I error rate at 10%).

## Results

**Figure 1:**
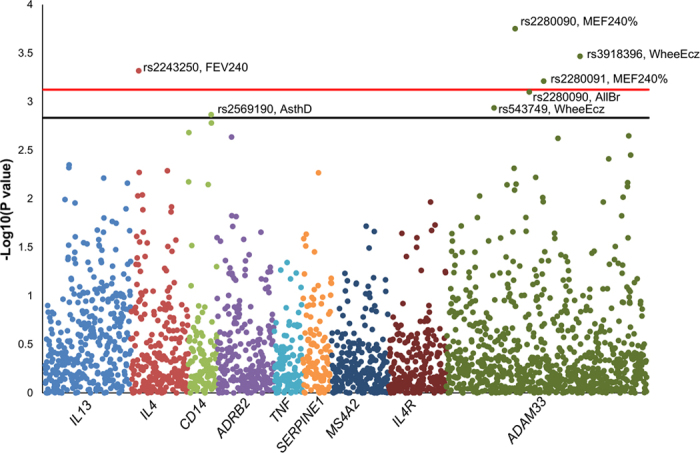
Manhattan plot of the PheWAS results. The plot is stratified by gene and, within each gene group, we report the results related to the phenotypic traits investigated. Red and black lines indicate the phenome-wide and the suggestive significance thresholds (p = 7.53*10^−4^ and p = 1.47*10^−3^, respectively). MEF240%: Mean Expiratory Flow after 240s of hypertonic saline inhalation with respect to the age- and ancestry-matched reference value; WheeEcz: wheezing and eczema comorbidity; FEV240: Forced Expiratory Flow Volume after 240s of hypertonic saline inhalation; AllBr: allergic bronchitis; AsthD: asthma diagnosis.

**Figure 2:**
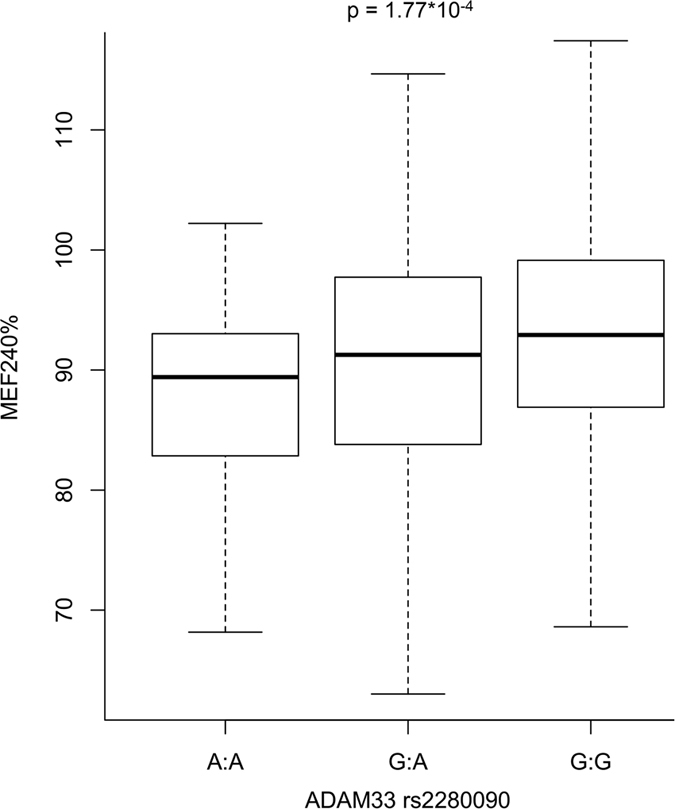
MEF240% (Mean Expiratory Flow after 240s of hypertonic saline inhalation with respect to the age- and ancestry-matched reference value) distribution across *ADAM33* rs2280090 genotypes.

**Figure 3:**
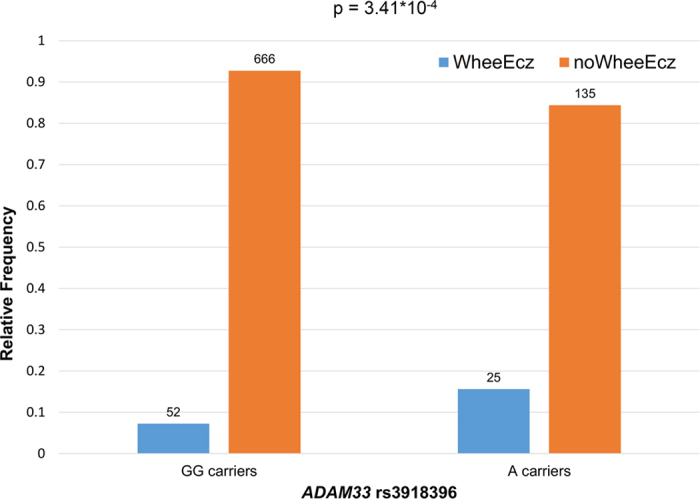
Wheezing and Eczema comorbidity with respect to *ADAM33* rs3918396. We graphically reported a dominant genetic model (GG carriers vs. A carriers) since our cohort only included 8 subjects with *ADAM33* rs3918396 AA genotype.

**Figure 4:**
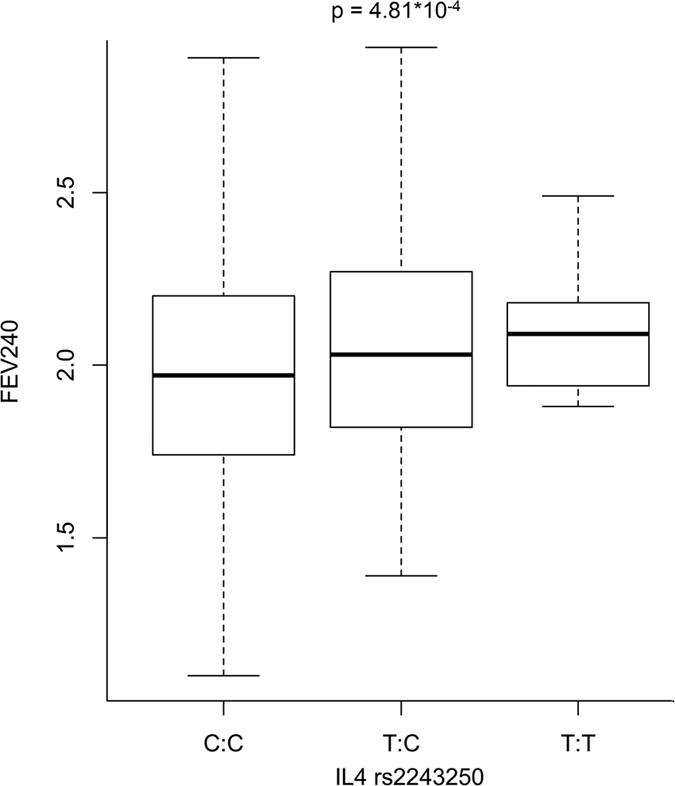
FEV240 (Forced Expiratory Flow Volume after 240s of hypertonic saline inhalation) distribution across *IL4* rs2243250 genotypes.

The Manhattan plot reported in Fig. 1 summarizes the results of our ISAAC-based PheWAS. PWS and suggestive findings were observed for SNPs located in *ADAM33*, *IL4*, and *CD14*. The top finding was the association between *ADAM33* rs2280090 and the ratio of Mean Expiratory Flow after 240s of hypertonic saline inhalation with respect to the age- and ancestry-matched reference value (MEF240%; z = −3.77, p = 1.77*10^−4^). MEF240% was also PWS associated with *ADAM33* rs2280091 (z = −3.44, p = 6.15*10^−4^) due to the high linkage disequilibrium (LD) between the two *ADAM33* variants (r^2^ = 98%; Supplemental Fig. 1). The *ADAM33* rs2280090 A allele is associated with lower MEF240% (Fig. 2): MEF240%_median_ = 89% for AA genotype; MEF240% median = 91% for AG genotype; MEF240%_median_ = 93% for GG genotype. *ADAM33* rs2280090 also showed a suggestive association for allergic bronchitis: individuals with *ADAM33* rs2280090*A allele have increased risk to have an allergic bronchitis (z = 3.36, p = 7.94*10^−4^). The second strongest PWS result was observed between *ADAM33* rs3918396 and the wheezing and eczema comorbidity (z = 3.60; p = 3.41*10^−4^). Individuals with *ADAM33* rs3918396 A allele have an increased risk to present wheezing and eczema comorbidity with respect to carriers of GG genotype (16% vs. 7%, respectively; Fig. 3). Wheezing and eczema comorbidity was also associated with another *ADAM33* variant (rs543749, z = 3.259, p = 1.16*10^−3^) in high LD with rs3918396 (r^2^ = 66%). The only PWS association observed outside *ADAM33* locus was between *IL4* rs2243250 and Forced Expiratory Flow Volume after 240s of hypertonic saline inhalation (FEV240; z = 3.51, p = 4.81*10^−4^). *IL4* rs2243250 T allele was associated with increased FEV240 (Fig. 4): FEV240_median_ = 1.97 for CC genotype; FEV240 median = 1.99 for TC genotype; FEV240_median_ = 2.03 for TT genotype. We observed an additional suggestive association between *CD14* rs2569190: individuals with *CD14* rs2569190 G allele have an increased risk of having an asthma diagnosis (z = 3.214; p = 1.36*10^−3^).

## Discussion

The present study provided novel information regarding the genetics of allergic respiratory diseases in Turkish children. To our knowledge, no genetic study has been previously conducted on large Turkish cohorts and no PheWAS has been performed to understand the role of immunogenes in allergic respiratory diseases. Our data indicated that risk alleles located in *ADAM33* rs2280090 and rs3918396, *IL4* rs2243250 and *CD14* rs2569190 are associated in allergy, asthma, and other related phenotypic traits such as wheezing and atopy.

The strongest results were observed for variants located in *ADAM33* gene. Although *ADAM33* was not yet confirmed by large GWAS as risk locus for allergy and asthma, polymorphisms in this gene are associated with bronchial hyperresponsiveness (BHR), asthma, and allergy across different populations^32^. Numerous experimental evidences support its involvement in allergy and lung function^8^. Human and animal studies indicated that ADAM33 is also critically involved in inflammatory lung diseases: it is upregulated during acute or chronic lung inflammation^33^. Even though this functional link between *ADAM33* and allergic airway inflammation, its role in the pathophysiology of allergic respiratory diseases is still to be clarified. Our PheWAS indicated that *ADAM33* is a risk locus with multiple variants associated with allergy-related phenotypes: MEF240%, allergic bronchitis, and wheezing-eczema comorbidity. MEF240% is a parameter of the hypertonic saline challenge test recommended to assess bronchial hyperresponsiveness (BHR) by ISAAC^34^. *ADAM33* rs2280090 and rs2280091 were associated with a reduced MEF240% and an increased risk of developing allergic bronchitis. Both of these phenotypes are closely related to BHR which is strongly related to allergic inflammatory processes^35^. Accordingly, our data agree with previous experimental studies, confirming that genetic variation at *ADAM33* locus is involved in the predisposition to allergic inflammation and lung function. We also observed that other *ADAM33* variants (rs3918396 and rs543749) are associated with wheezing and eczema comorbidity. In our previous study on Turkish children^26^, subjects with current wheezing had a two-fold increased risk of eczema than the other participants. It was hypothesized that epicutaneous sensitization with subsequent migration of sensitized T cells into the airways and nose can cause symptoms related to asthma and allergic rhinitis and consequently contribute to comorbidity in allergic disease^36^. Our genetic results indicated that *ADAM33* might be involved in the molecular processes associated with the comorbidity of wheezing and eczema. This is supported by the fact that *ADAM33* is a tissue susceptibility factor involved in epithelial/epidermal barrier function and remodeling and these mechanisms are relevant in both atopic dermatitis and asthma^37^. Furthermore, a recent genome-wide study revealed that eczema loci increase the risk of atopic march (i.e., the sequential progression of different allergic conditions), suggesting that eczema and allergic respiratory diseases share a consistent genetic component^38^.

Two further associations were observed for *IL4* and *CD14* variants. *IL4* rs2243250 was associated with increased FEV240. Many gene-candidate studies reported the association of *IL4* variants with pediatric asthma and allergy^39^ and a recent GWAS of atopic march identified a risk allele in *IL4* region^38^. Functional experiments demonstrated that IL-4 is a Th2 cytokine that plays a key role in inflammation-induced airway remodeling^40^. The association of *IL4* rs2243250 with increased FEV240 demonstrated that variants in this locus predispose to alterations of lung function in relation to pulmonary stress. We also found a suggestive evidence supporting the association of *CD14* rs2569190 with asthma diagnosis. This gene codifies a co-receptor for the toll-like receptor that have an high specificity for lipopolysaccharides and together with TLR4 forms a complex that activates the innate immune system^41^. A recent systematic review analysis hypothesized a three-way interaction between *CD14* polymorphisms, microbial exposures and the age of exposure in relation to allergic diseases^42^. Endotoxin and microbial exposure may modulate the risk of allergy associated to *CD14* rs2569190 alleles^43^. Our data confirmed the role of *CD14* in the predisposition to asthma in Turkish children.

In conclusion, this study provided novel data regarding the role of immunogenetics in relation to asthma- and allergy-related phenotypes. Our main results showed significant association for quantitative traits (i.e., MEF240%, FEV240) or endophenotypes (e.g., wheezing-eczema comorbidity), but not in the main diagnostic phenotypes (e.g., asthma diagnosis and BHR). This confirms that PheWAS (the investigation of numerous phenotypes with respect to known risk loci) are a powerful tool to dissect the molecular mechanisms involved in the predisposition to complex traits. Specifically, the genes involved in immune response appeared to play a relevant role in the processes linked to allergic airway inflammation, lung function, and eczema-wheezing comorbidity. Further investigations of large cohorts, such Mendelian randomization studies^44^ and polygenic risk score analysis^45^, can dissect how the genetics of lower lung function affects the risk of allergic disease.

## Additional Information

**How to cite this article**: Karaca, S. *et al*. Allergy-specific Phenome-Wide Association Study for Immunogenes in Turkish Children. *Sci. Rep*. **6**, 33152; doi: 10.1038/srep33152 (2016).

## Supplementary Material

Supplementary Information

## Figures and Tables

**Table 1:** Main characteristics of the study population.

Characteristics	Study Population (n = 974)
Age (Years), Median (Range)	11 (9–16)
Sex (Female), n (%)	473 (49)
Ever Wheezing, n (%)	583 (60)
Asthma diagnosis, n (%)	71 (7)
Bronchitis diagnosis, n (%)	349 (37)
Bronchial Hyperresponsiveness, n (%)	261 (23)
Allergic bronchitis diagnosis, n (%)	119 (13)
Allergic rhinoconjunctivitis, n (%)	74 (11)
Positive skin prick test, n (%)	215 (22)
