# Supplementary material for: Allergy-specific Phenome-Wide Association Study for Immunogenes in Turkish Children
Source: Sci Rep. 2016 Sep 14;6:33152. doi: 10.1038/srep33152 (PMC5021980; doi:10.1038/srep33152)

**Allergy-specific Phenome-Wide Association Study for Immunogenes in Turkish Children**

Şefayet Karaca^1^, Ersoy Civelek^2^, Mehmet Karaca^3^, Ümit M. Şahiner^4^, Riza K. Ozgul^5^, Can N. Kocabaş^2^, Renato Polimanti^6*^, Bülent E. Şekerel^4*^

^1^Aksaray University, Faculty of Health Science, Aksaray, Turkey

^2^Ankara Child Health and Diseases Hematology Oncology Research Hospital, Pediatric Allergy and Immunology Clinic, Ankara, Turkey

^3^Aksaray University, Faculty of Science and Arts, Department of Biology, Aksaray, Turkey

^4^Hacettepe University, Faculty of Medicine, Pediatric Allergy and Asthma Unit, Ankara, Turkey

^5^Hacettepe University, Faculty of Medicine, Pediatrics Department, Unit of Metabolism and Institute of Child Health, Ankara, Turkey

^6^Department of Psychiatry, Yale University School of Medicine and VA CT Healthcare Center, West Haven, CT, United States

*These authors share the senior authorship

**Running Ttile**: PheWAS for immunogenes in Turkish children

**Corresponding Author**: Sefayet Karaca, PhD. Aksaray University, School of Health Science, Aksaray, Turkey. Phone: +905325068202, Fax: +903822882545, E-mail: [skaraca@aksaray.edu.tr](mailto:skaraca@aksaray.edu.tr)

**Supplemental Table 1:** Details of phenotypes tested.

| **Category** | **Phenotypic trait** | **Type** | **N** |
| --- | --- | --- | --- |
| Blood test | serum IgE level | Binary | 895 |
|  | % eosinophils | Quant | 920 |
|  | % lymphocytes | Quant | 925 |
|  | % neutrophils | Quant | 921 |
|  | > 4% eosinophils | Binary | 920 |
|  | 100 kU/L of serum IgE level | Quant | 895 |
|  | absolute eosinophil count | Quant | 916 |
|  | absolute lymphocyte count | Quant | 733 |
|  | absolute neutrophil count | Quant | 731 |
|  | White blood cell count | Quant | 922 |
|  | Hematocrite level | Quant | 926 |
|  | Hemoglobine concetration | Quant | 925 |
|  | Mean Corpuscular Volume | Quant | 910 |
|  | Platelet count | Quant | 926 |
|  | Red blood cell distribution width | Quant | 910 |
| Bronchial Challenge Test | % FEV120 compared to healthy individuals | Quant | 864 |
|  | % FEV240 compared to healthy individuals | Quant | 801 |
|  | % FEV30 compared to healthy individuals | Quant | 925 |
|  | % FEV480 compared to healthy individuals | Quant | 717 |
|  | % FEV60 compared to healthy individuals | Quant | 903 |
|  | % FEVini compared to healthy individuals | Quant | 925 |
|  | % FEVpost compared to healthy individuals | Quant | 921 |
|  | % MEF120 compared to healthy individuals | Quant | 865 |
|  | % MEF240 compared to healthy individuals | Quant | 800 |
|  | % MEF30 compared to healthy individuals | Quant | 925 |
|  | % MEF480 compared to healthy individuals | Quant | 717 |
|  | % MEF60 compared to healthy individuals | Quant | 904 |
|  | % MEFini compared to healthy individuals | Quant | 925 |
|  | Bronchial hyperresponsiveness (BCT Result) | Binary | 923 |
|  | FEV after 120s of hipertonic saline inhalation | Quant | 864 |
|  | FEV after 240s of hipertonic saline inhalation | Quant | 801 |
|  | FEV after 30s of hipertonic saline inhalation | Quant | 925 |
|  | FEV after 480s of hipertonic saline inhalation | Quant | 717 |
|  | FEV after 60s of hipertonic saline inhalation | Quant | 903 |
|  | FEV after bronchodilator drug inhaltion subsequently to BTC | Quant | 921 |
|  | Forced Expiratory Flow Volume (FEV) before Bronchial Challenge Test (BCT) | Quant | 925 |
|  | Initial FEV during BCT | Quant | 925 |
|  | Initial MEF during BCT | Quant | 925 |
|  | Mean expratory Flow (MEF) before Bronchial Challenge Test (BCT) | Quant | 925 |
|  | MEF after 120s of hipertonic saline inhalation | Quant | 865 |
|  | MEF after 240s of hipertonic saline inhalation | Quant | 800 |
|  | MEF after 30s of hipertonic saline inhalation | Quant | 925 |
|  | MEF after 480s of hipertonic saline inhalation | Quant | 717 |
|  | MEF after 60s of hipertonic saline inhalation | Quant | 904 |
|  | MEF after bronchodilator drug inhaltion subsequently to BTC | Quant | 922 |
|  | MEFpost compared to healthy individuals | Quant | 922 |
| Medical Evaluation | 4 or more Wheezing | Binary | 970 |
|  | Allergic bronchitis diagnosis at least once | Binary | 941 |
|  | Allergic bronchitis diagnosis at least twice | Binary | 941 |
|  | Allergic rhinoconjuctivitis | Binary | 666 |
|  | Asthma and Wheezing | Quant | 974 |
|  | Asthma diagnosis at least once | Binary | 936 |
|  | Asthma diagnosis at least once and BHR positive | Binary | 923 |
|  | Asthma diagnosis at least twice | Quant | 936 |
|  | Asthma severity | Quant | 323 |
|  | Asthma, asthmatic bronchitis, or allergic bronchitis diagnosis | Quant | 974 |
|  | Asthmatic bronchitis diagnosis at least once | Binary | 950 |
|  | Asthmatic bronchitis diagnosis at least twice | Quant | 950 |
|  | At least 2 wheezing episodes | Binary | 970 |
|  | At least 32 wheezing episodes | Binary | 970 |
|  | Atopic and non-Atopic Wheezing | Quant | 966 |
|  | Atopic Wheezing (Y/N) | Binary | 966 |
|  | Birth time (premature vs. mature) | Binary | 902 |
|  | Birth weight (<2,500 grams) | Binary | 850 |
|  | Bronchitis diagnosis at least once | Binary | 950 |
|  | Bronchitis diagnosis at least twice | Quant | 950 |
|  | Current Atopic Disease | Binary | 974 |
|  | Current Atopic Symptoms | Binary | 974 |
|  | Current Eczema | Binary | 974 |
|  | Current Eczema with flexural dermatitis | Binary | 974 |
|  | Current Respiratory Symptoms | Binary | 974 |
|  | Current Wheezin and BHR positive | Binary | 923 |
|  | Current Wheezing | Binary | 974 |
|  | Current wheezing and asthma diagnosis at least once | Binary | 974 |
|  | Current Wheezing or Allergic Rhinoconjoctivitis Symptoms | Binary | 974 |
|  | Current wheezing, BHR positive, and asthma diagnosis at least once | Binary | 923 |
|  | diagnosis of asthma, asthmatic bronchitis, allergic bronchitis, or bronchitis at least once | Binary | 963 |
|  | diagnosis of asthma, asthmatic bronchitis, allergic bronchitis, or bronchitis at least twice | Binary | 963 |
|  | Ever allergic bronchitis diagnosis | Binary | 941 |
|  | Ever asthma diagnosis | Binary | 936 |
|  | Ever asthmatic bronchitis diagnosis | Binary | 950 |
|  | Ever atopic diseases | Quant | 974 |
|  | Ever bronchitis diagnosis | Binary | 950 |
|  | Ever Eczema | Binary | 974 |
|  | Ever Severe Wheezing episode | Binary | 974 |
|  | Ever Wheezing | Binary | 970 |
|  | Mild non-complicated Wheezing | Binary | 974 |
|  | Mild non-complicated Wheezing severity | Quant | 974 |
|  | Positive Physical Examination | Binary | 946 |
|  | Rhinitis and current wheezing status | Quant | 930 |
|  | Rhinitis atopic status | Quant | 924 |
|  | Rhinitis classification | Quant | 974 |
|  | Rhinoconjunctivitis | Binary | 930 |
|  | Waking up at night with wheezing | Quant | 565 |
|  | Waking up with wheezing | Binary | 565 |
|  | Wheezing (number) | Quant | 970 |
|  | Wheezing and Allergic rhinitis | Quant | 952 |
|  | Wheezing and Eczema | Quant | 955 |
|  | Wheezing and Rhinoconjunctivitis | Quant | 952 |
|  | Wheezing plus Eczema (Y/N) | Binary | 955 |
|  | Wheezing plus Rhinoconjunctivitis (Y/N) | Binary | 952 |
|  | Wheezing scale | Quant | 974 |
|  | Wheezing severity | Quant | 974 |
| 3 mm Skin test | Olea atopy | Binary | 960 |
|  | Alternaria atopy | Binary | 960 |
|  | Atopy (general) | Binary | 960 |
|  | Cat atopy | Binary | 960 |
|  | Cockroach atopy | Binary | 960 |
|  | Grass atopy | Binary | 960 |
|  | Horse atopy | Binary | 960 |
|  | House dust mite (DF) atopy | Binary | 960 |
|  | House dust mite (DP) atopy | Binary | 960 |
|  | Mite atopy | Binary | 960 |
|  | Pollen atopy | Binary | 960 |
|  | Tree atopy | Binary | 960 |
|  | Flexural dermatitis | Binary | 946 |

**Supplemental Table 2:** Details of the sequence-specific amplification primers.

| **SNP ID** | **F-Primer_Allel-FAM** | **F-Primer_Allel-HEX** | **R-Primer** |
| --- | --- | --- | --- |
| rs20541 | TGATGCTTTCGAAGTTTCAGTTGAACT | GATGCTTTCGAAGTTTCAGTTGAACC | GCCCAGTTTGTAAAGGACCTGCTCTTA |
| rs1295686 | GCAGGACAAAGAGGTCAGCACA | CAGGACAAAGAGGTCAGCACG | CGTCCCGGCCTCTGGCGTT |
| rs1800925 | CCTTTTCCTGCTCTTCCCTCG | GCCTTTTCCTGCTCTTCCCTCA | GGGTTTCTGGAGGACTTCTAGGAAA |
| rs1800629 | GGAGGCTGAACCCCGTCCT | GAGGCTGAACCCCGTCCC | GAGGCAATAGGTTTTGAGGGGCAT |
| rs1801275 | CCACCGCATGTACAAACTCCT | CCACCGCATGTACAAACTCCC | CGGCCCCCACCAGTGGCTA |
| rs1805015 | CACGGTGACTGGCTCAGGGA | ACGGTGACTGGCTCAGGGG | CCTGCTTACCGCAGCTTCAGCAA |
| rs2569190 | GCAGAATCCTTCCTGTTACGGT | GCAGAATCCTTCCTGTTACGGC | CCTTTCCTGGAAATATTGCAATGAAGGAT |
| rs511898 | GCCCTCCAAATCAGAAGAGACG | ATAGCCCTCCAAATCAGAAGAGACA | CTGGGACTCGAGGCCTGTGAAT |
| rs3918396 | GGGAGCAGAGGCAGCAGGAT | GGAGCAGAGGCAGCAGGAC | TCCCCGCAGACCATGACACCTT |
| rs2280091 | GGGCGGCGTTCACCCCAT | GGGCGGCGTTCACCCCAC | GCCAGGGCTGTCCAGTGGCT |
| rs2787094 | GAGTCCACACTCCCCTGC | CTGAGTCCACACTCCCCTGG | TATGTTTGTTTGCAGAGGCCAGCCA |
| rs543749 | AGGAGGATATGTTGTCCCCTAAG | GAGGAGGATATGTTGTCCCCTAAT | TGCAGCCTGGGGCCCCAGT |
| rs2280090 | CCCCACAGCCACTGGACAGT | CCCACAGCCACTGGACAGC | CCTCACTCACCCAGGGGCCA |
| rs2243250 | CACCTAAACTTGGGAGAACATTGTC | AACACCTAAACTTGGGAGAACATTGTT | GCAGAATAACAGGCAGACTCTCCTA |
| rs2070874 | GTCGATTTGCAGTGACAATGTGAGA | CGATTTGCAGTGACAATGTGAGG | GCATCGTTAGCTTCTCCTGATAAACTAAT |
| rs6127096 | GGGATTCAAACGGCAAGGAGGA | CAAACGGCAAGGAGGG | CCTAGAATGGTGAGCTCTGCCCA |
| rs1042713 | GCCTTCTTGCTGGCACCCAATA | CCTTCTTGCTGGCACCCAATG | CGTGGTCCGGCGCATGGCTT |
| rs1042714 | GACCACGACGTCACGCAGC | GACCACGACGTCACGCAGG | ACCACCCACACCTCGTCCCTTT |
| rs569108 | ATTCTTATAAATCAATGGGAGGAGACATTT | CTTATAAATCAATGGGAGGAGACATTC | GTTCCAGAGGATCGTGTTTATGAAGAATT |
| rs1441586 | ATTTCATATTAGTCTTTATTTAGTAGACTTCTC | TCATATTAGTCTTTATTTAGTAGACTTCTT | GGCTGATTAAGATCAACAGGCTTTTACTT |
| rs1799768 | CCGATGATACACGGCTGACT | CCGATGATACACGGCTGACC | CAGAGAGAGTCTGGACACGTGG |

**Supplemental Table 3**: Details of SNPs tested. CHR: Chromosome, BP: Base pair, HWpval: Hardy-Weinberg equilibrium p value (exact test), %Geno: genotype call rate, MAF: Minor Allele frequency.

| **rsID** | **CHR** | **BP** (hg38) | **Gene** | **HWpval** | **%Geno** | **MAF** | **Alleles** |
| --- | --- | --- | --- | --- | --- | --- | --- |
| rs1800925 | 5 | 132657117 | *IL13* | 0.9038 | 98.8 | 0.21 | C>T |
| rs1295686 | 5 | 132660151 |  | 0.356 | 96.8 | 0.249 | G>A |
| rs20541 | 5 | 132660272 |  | 0.4077 | 94.5 | 0.235 | C>T |
| rs2243250 | 5 | 132673462 | *IL4* | 0.4131 | 99.4 | 0.129 | C>T |
| rs2070874 | 5 | 132674018 |  | 0.4867 | 95.7 | 0.12 | C>T |
| rs2569190 | 5 | 140633331 | *CD14* | 0.2355 | 94.7 | 0.46 | A>G |
| rs1042713 | 5 | 148826877 | *ADRB2* | 0.8103 | 98.9 | 0.397 | G>A |
| rs1042714 | 5 | 148826910 |  | 0.8155 | 99.2 | 0.306 | C>G |
| rs1800629 | 6 | 31575254 | *TNF* | 1 | 99.1 | 0.078 | G>A |
| rs1799768 | 7 | 100769706 | *SERPINE1* | 0.214 | 95 | 0.486 | G>A |
| rs1441586 | 11 | 60088555 | *MS4A2* | 0.8058 | 98.8 | 0.477 | C>T |
| rs569108 | 11 | 60095631 |  | 1 | 95.4 | 0.028 | T>C |
| rs1805015 | 16 | 27362859 | *IL4R* | 0.4829 | 99.6 | 0.112 | T>C |
| rs1801275 | 16 | 27363079 |  | 0.4182 | 98.4 | 0.146 | A>G |
| rs2787094 | 20 | 3668514 | *ADAM33* | 0.3094 | 98.3 | 0.252 | G>C |
| rs543749 | 20 | 3669032 |  | 0.988 | 99.7 | 0.138 | G>T |
| rs2280090 | 20 | 3669558 |  | 0.8536 | 99.6 | 0.21 | G>A |
| rs2280091 | 20 | 3669587 |  | 0.9251 | 94.9 | 0.211 | A>G |
| rs3918396 | 20 | 3671118 |  | 1 | 96.7 | 0.095 | G>A |
| rs6127096 | 20 | 3671560 |  | 0.7947 | 98.7 | 0.146 | G>A |
| rs511898 | 20 | 3674438 |  | 0.5695 | 97.8 | 0.405 | C>T |

**Supplemental Figure 1:** LD structure of the immunogenes investigated. The intensity of the box shading is proportional to the strength of the LD (r^2^) for the marker pair, which is also indicated as a percentage of r^2^ within each box.


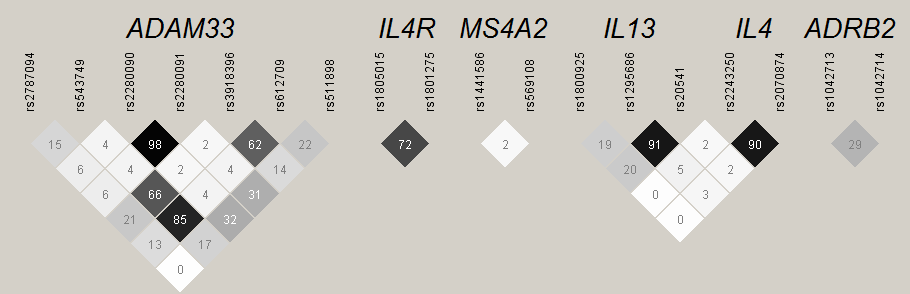

Supplement: Supplementary Information [file srep33152-s1.doc]
